# Transcriptional analysis of early lineage commitment in human embryonic stem cells

**DOI:** 10.1186/1471-213X-7-12

**Published:** 2007-03-02

**Authors:** Andrew L Laslett, Sean Grimmond, Brooke Gardiner, Lincon Stamp, Adelia Lin, Susan M Hawes, Sam Wormald, David Nikolic-Paterson, David Haylock, Martin F Pera

**Affiliations:** 1 Monash Institute of Medical Research, Monash University and the Australian Stem Cell Centre, Clayton, Victoria Australia; 2 Institute of Molecular Biosciences, University of Queensland, Saint Lucia, Queensland, Australia; 3 The Walter and Eliza Hall Institute of Medical Research and the Cooperative Research Centre for Cellular Growth Factors, Parkville, Victoria, Australia; 4 Department of Medicine, Monash Medical Centre, Clayton, Victoria, Australia; 5 Peter Macallum Cancer Centre, Melbourne, Victoria, Australian and the Australian Stem Cell Centre, Clayton, Victoria, Australia; 6 Center for Stem Cell and Regenerative Medicine, Keck School of Medicine, University of Southern California, Los Angeles, CA, USA

## Abstract

**Background:**

The mechanisms responsible for the maintenance of pluripotency in human embryonic stem cells, and those that drive their commitment into particular differentiation lineages, are poorly understood. In fact, even our knowledge of the phenotype of hESC is limited, because the immunological and molecular criteria presently used to define this phenotype describe the properties of a heterogeneous population of cells.

**Results:**

We used a novel approach combining immunological and transcriptional analysis (immunotranscriptional profiling) to compare gene expression in hESC populations at very early stages of differentiation. Immunotranscriptional profiling enabled us to identify novel markers of stem cells and their differentiated progeny, as well as novel potential regulators of hESC commitment and differentiation. The data show clearly that genes associated with the pluripotent state are downregulated in a coordinated fashion, and that they are co-expressed with lineage specific transcription factors in a continuum during the early stages of stem cell differentiation.

**Conclusion:**

These findings, that show that maintenance of pluripotency and lineage commitment are dynamic, interactive processes in hESC cultures, have important practical implications for propagation and directed differentiation of these cells, and for the interpretation of mechanistic studies of hESC renewal and commitment. Since embryonic stem cells at defined stages of commitment can be isolated in large numbers by immunological means, they provide a powerful model for studying molecular genetics of stem cell commitment in the embryo.

## Background

The first seven years of research on human embryonic stem cells (hESC) have led to significant advances in our ability to maintain and manipulate these fascinating cultured cell lines [1-3]. The initial reports of the derivation of pluripotent stem cells from the human blastocyst [4,5] have been abundantly confirmed, technology for the maintenance and manipulation of hESC has been successfully disseminated around the world, and there have been improvements to the culture system used in the first derivations. The differentiation in vitro of hESC into a variety of tissue types of enormous potential significance to research and medicine, including neural tissue, blood, cardiac muscle, and many others, has been reported, and the first studies showing proof of principle of the application of hESC-derived neural cells in preclinical animal models of disease have recently been published [6,7].

While this record is impressive, very significant challenges remain ahead if hESC are actually going to fulfill their potential. The reality is that even our basic understanding of the phenotype of human pluripotent stem cells is limited. hESC are characterized by their immunological profile, by transcriptional analysis, and by biological assay of their capability for self-renewal and multilineage differentiation. Most work carried out on hESC has made the tacit assumption that the canonical hESC phenotype-a cell positive for specific surface antigens (SSEA-3, SSEA-4, TRA-1–60, CD9), expressing genes specific to pluripotent cells (e.g., Oct-4, nanog), and capable of indefinite renewal and differentiation into derivatives of all three embryonic germ layers-essentially describes a single discrete cellular entity. However, the canonical description of the phenotype of the hESC in fact describes the properties of a heterogeneous population of cells, some of which have embarked on the pathway to differentiation. Because of this, and because the early stages of hESC commitment and differentiation are largely uncharted, present studies at the cellular, molecular and biochemical level, which treat hESC cultures as a homogeneous population of cells, are capable of providing only limited insight into the control of stem cell renewal and differentiation. In particular, the numerous studies of the hESC transcriptome and proteome, [8-19] which generally have compared hESC populations grown under conditions that support renewal to cultures undergoing overt differentiation, have produced a molecular blueprint of the pluripotent state, but this blueprint is limited in its resolution due to the inherent complexity of the cell populations under comparison.

The structure of stem cell differentiation hierarchies in general, and that of hESC in particular, is often depicted as a series of binary choices between alternate and discrete cell states, driven by a serial cascade of expression of specific transcription factors. However, other data indicate that for pluripotent stem cells at least, the early progression through a differentiation hierarchy is in fact a continuum that may be reversibly traversed [20]. In fact, emerging concepts regarding cell fate choice in the preimplantation mouse embryo support a less rigid interpretation of the process of lineage commitment. A newer model [21] depicts the formation of three specific lineages of the mammalian periimplantation embryo, inner cell mass, trophectoderm, and extraembryonic endoderm, not as a sequence of binary decisions, but as the result of a dynamic interplay of expression of a network of particular regulatory genes. Specifically, networks of key transcriptional regulators, including Oct-4, nanog, cdx-2 and GATA -4 and -6, interact in a spatially restricted fashion in the preimplantation embryo to determine fate, rather than acting in a sequential mode, as recently illustrated for commitment to the trophectoderm lineage [22,23]. These findings imply that in early development, the process of lineage choice begins early, before overt loss of all stem cell maintenance factors, and occurs through a set of opposing reciprocal interactions between key transcription factors. These concepts are reminiscent of the model of lineage priming, derived from studies of hematopoiesis, in which expression of genes characteristic of multiple differentiation lineages is observed in stem or progenitor cells that have not yet undertaken overt commitment [24-26]. A recent study [27] of the transcriptional regulatory circuits in hESC predicts that cell fate is the result of a dynamic interplay between key regulatory factors, and that alterations in stoichiometry between these factors lead to global changes in gene expression and ultimately cell commitment to specific lineages. Furthermore, this study highlighted a key role for pluripotency genes in the suppression of the expression of many lineage specific transcription factors.

In this study we sought to analyse the early stages in hESC differentiation through an approach we call immunotranscriptional profiling. We used flow cytometry to fractionate hESC populations grown under conditions that support stem cell renewal on the basis of their levels of expression of two surface markers. Following fractionation, each population was subjected to transcriptome analysis via microarray. Critical findings were confirmed by low density array quantitative reverse transcriptase polymerase chain reaction (QRT-PCR). The results identify new markers of the pluripotent state and potential paracrine regulators of cell fate, and they provide evidence for a continuum of expression of pluripotency genes and lineage specific transcription factors across the population.

## Results

### Antibodies to epitopes expressed on the surface of primate pluripotent stem cells reveal heterogeneity in hESC cultures

**Figure 1:**
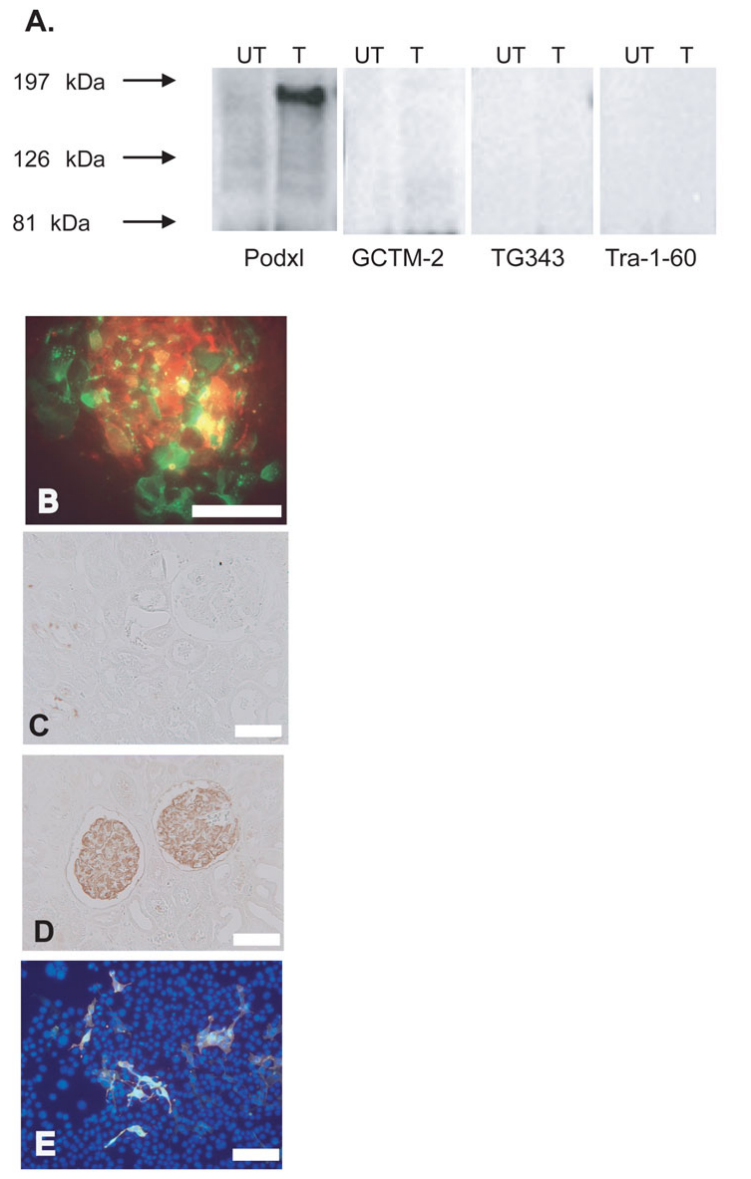
Reactivity of the hybridomas GCTM-2 and TG30 used to fractionate hESC by flow cytometry. A, Protein lysate from M15 cells transfected (T) with the full-length human podocalyxin construct, pcDNA3/Podxl, shows immunoreactivity to podocalyxin antibodies (PHM5). In contrast, no immunoreactivity was observed with GCTM-2, TG343 or TRA 1–60 antibodies, nor in the untransfected (UT) cells. B, Indirect immunofluorescent staining of hESC for podocalyxin (green) and GCTM-2 (red). C, Human kidney stained for GCTM-2. D, Human kidney stained for podocalyxin. E, Mouse STO cells transfected with human CD9 cDNA stained by indirect immunofluorescence for CD9 (red) and TG30 (green) and DAPI (blue). CD9 and TG30 staining is entirely coincident. Bar in B-E = 100 μm.

In our investigations we used three monoclonal antibodies recognising canonical markers of hESC. The first was the monoclonal antibody GCTM-2, which recognises an epitope on the protein core of a high molecular weight pericellular matrix keratan sulphate/chondroitin sulphate proteoglycan [28]. A recent study identified the GCTM-2 antigen as the CD34 related sialomucin podocalyxin [29]. To assess this conclusion, which was based primarily on copurification of the GCTM-2 antigen and podocalyxin on lectin affinity columns, we transfected a mouse kidney cell line with human podocalyxin cDNA. Anti-podocalyxin antibodies detected a band of the appropriate molecular weight in immunoblots of extracts, but there was no reaction with either GCTM-2, TG343 (another antibody reactive with a distinct epitope on the core protein of the proteoglycan, [30]) or the antibody TRA-1–60, which reacts with a carbohydrate epitope on the same molecule (Figure 1a). While both GCTM-2 and anti-podocalyxin antibodies stained cells in hESC cultures, the populations stained were distinct (Figure 1b), and in the human kidney, GCTM-2 stained tubular epithelium weakly whilst anti-podocalyxin antibodies stained podocytes as expected (Figure 1c–d). Thus it is not clear that the GCTM-2 antigen and podocalyxin represent the same molecular entity; it is however apparent that podocalyxin antibodies recognise cells that GCTM-2 does not.

We also used monoclonal antibody TG30, produced by immunisation of mice with a partially purified preparation of the GCTM-2 antigen. TG30 reacts with a cell surface epitope on a 25 kDa protein. This epitope was identified as the tetraspannin protein CD9 following transfection of mouse STO cells with a human CD9 cDNA clone and demonstration of reactivity of the transfected cells with TG30 (Figure 1e). Others have reported expression of CD9 in hESC [14]. Finally we used a monoclonal antibody against the transcription factor Oct3/4, a molecule with long established function in the maintenance of pluripotency in mouse ES cells [31]; recent data strongly supports a similar role for this transcription factor in hESC [32,33].

**Figure 2:**
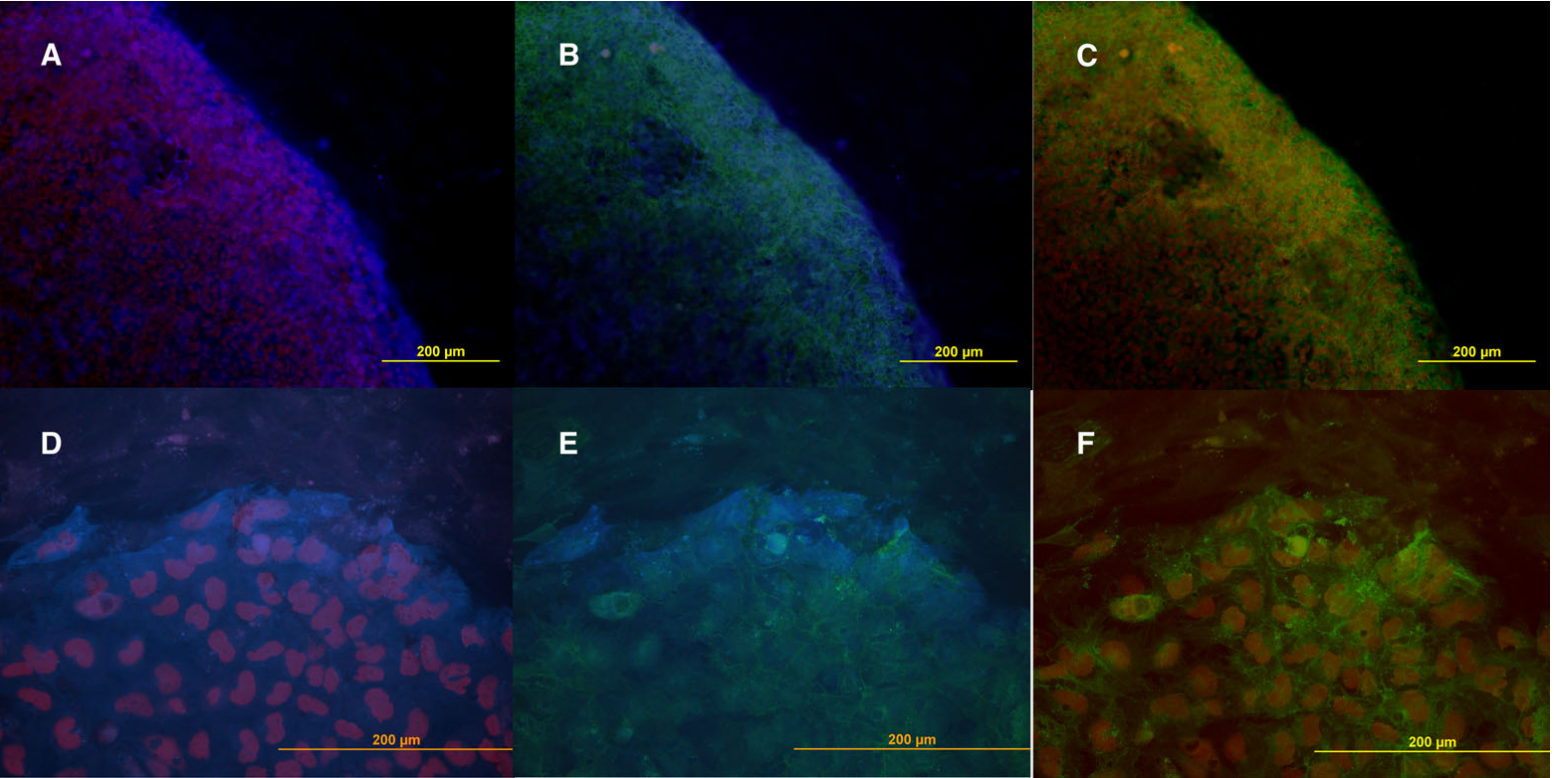
Triple label indirect immunofluorescence examination of stem cell marker expression in growing colonies of hESC. A&D GCTM-2 (blue) and Oct-4 (red). B&E GCTM-2 (blue) and CD9 (green). C&F CD9 (green) and Oct-4 (red). Scale bar = 200 μM.

HESC grown in serum-containing medium in the presence of mouse embryonic fibroblast feeder cell support were examined by double and triple label indirect immunofluorescence (Figure 2). The cultures were heterogeneous in their expression of these surface markers. In healthy growing colonies prior to overt cellular differentiation, only those cells at the edge of the colony expressed all three markers. Further in towards the colony centre, GCTM-2 staining was reduced, but CD9 and Oct-4 staining remained high. As differentiation proceeded, the interior of the colony became negative for all of these markers but the outer rim remained positive.

### Levels of expression of stem cell surface antigens GCTM-2 and CD9 correlate with levels of Oct-4 expression

**Figure 3:**
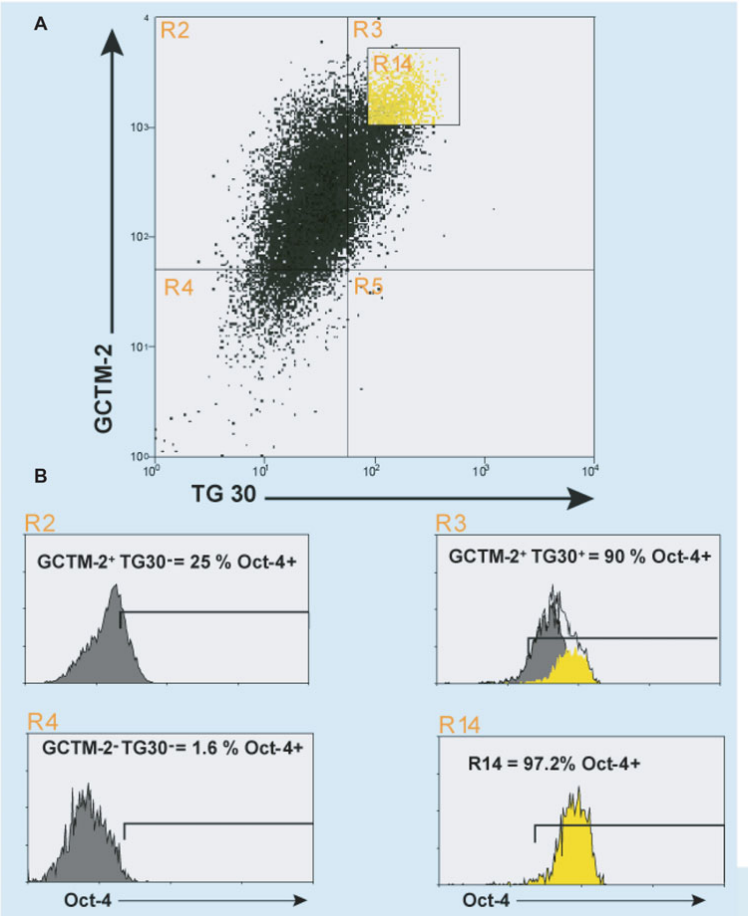
Combined flow cytometric analysis of hESC for GCTM-2, TG30 and Oct-4. Gates are set relative to isotype controls. A, yellow represents cells with high staining intensity for both GCTM-2 and TG30 (R14). B, Percentages of cells staining for Oct-4 from regions R2, R3, R4 and R14 of figure 3A.

The results above were suggestive of a gradient of antigen expression in growing colonies of hESC, but these indirect immunofluorescence data were qualitative only. The heterogeneity of antibody staining apparent on indirect immunofluorescence prompted us to carry out quantitative analysis of antigen expression by flow cytometry. We sought to establish the relationship between staining levels for the two surface antigens and intracellular levels of Oct-4 protein. Flow cytometric analysis of healthy growing colonies of hESC prior to overt differentiation showed that there was a gradient in staining levels for the surface antigens; most cells expressed both antigens, but some expressed only one or the other (Figure 3, top panel). There was a strong relationship between the levels of expression of Oct-4 and the intensity of double surface staining for GCTM-2 and CD9 (Figure 3, lower panel). Thus, the cell population expressing the highest level of both surface markers also expressed the highest level of Oct-4 positivity.

### Microarray analysis of gene expression in immunologically defined subpopulations of hESC shows coordinated regulation of gene expression and surface markers

**Figure 4:**
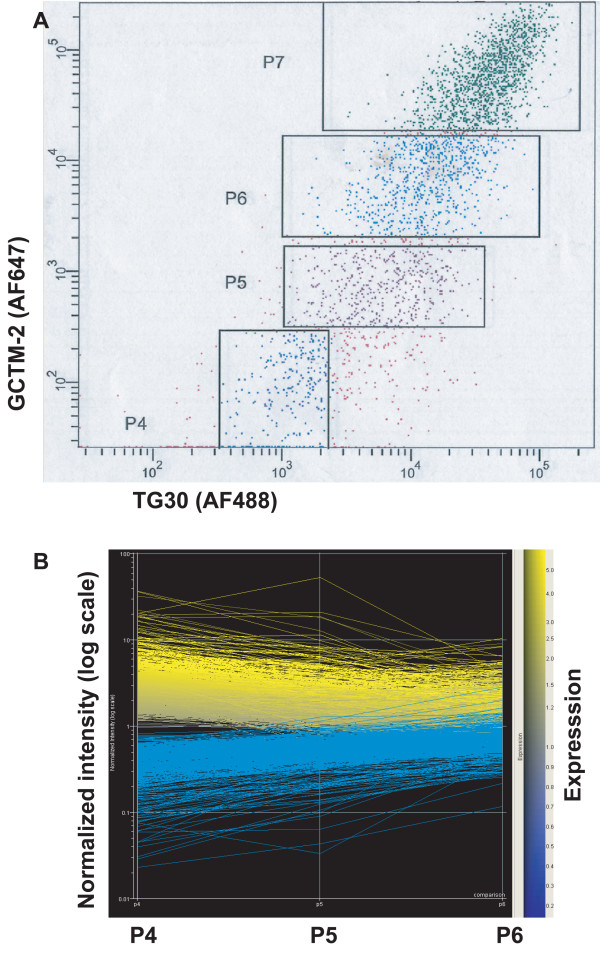
FACS of hESC for array analysis. Gates are set relative to isotype controls. A, hESC were separated by FACS according to staining intensity for GCTM-2 and TG30 (CD9) into 4 populations; P4 (GCTM-2^-^CD9^-^), P5 (GCTM-2^LOW^CD9^LOW^), P6 (GCTM-2^MID^CD9^MID^) and P7 (GCTM-2^HIGH^CD9^HIGH^). B, heat map depicting normalized intensity of gene expression for genes from P7 V P4 experiment with a B stat greater than zero.

The relationship between levels of expression of two stem cell markers and the transcription factor Oct-4 suggested the possibility that there might be an overall gradient of expression of stem cell markers in the hESC populations. To assess this we used microarray analysis to examine global gene expression patterns in immunologically defined subpopulations of hESC. Cells were sorted into four separate populations (Figure 4a), according to their expression of both surface markers, RNA was isolated, cDNA prepared, subjected to linear amplification, and then analysed by Compugen microarray.

We carried out global analysis of those genes showing significantly different levels of expression in P7 (GCTM-2^HIGH^CD9^HIGH^) versus P4 (GCTM-2^-^CD9^-^), P5 (GCTM-2^LOW^CD9^LOW^) and P6 (GCTM-2^MID^CD9^MID^). A two-fold difference in expression levels and a B-statistic greater than zero were the criteria used to identify significant changes. The detection rates were: P7 versus P6, 12,497/19,317 genes present; P7 versus P5, 12,094/19,317 genes present; and P7 versus P4, 15,059/19,317 present.

Most genes whose expression levels changed significantly showed consistent increases or decreases across the overall population, and only a small minority fluctuated up and down (Figure 4b). In all, the total number of significant changes in gene expression comparing P7 with the other populations were: P7 versus P6, 293 genes, with 264 higher in P7; P7 versus P5, 392 genes, 271 with higher in P7; P7 versus P4, 1051 genes, with 672 higher in P7. Inspection of the entire dataset (GEO Accession Number GSE4020) shows that changes in the expression of genes associated with the pluripotent state and those associated with early commitment events occur dynamically before the extinction of surface marker expression.

The comparison of the two most closely related subpopulations of cells, P7 and P6 [see Additional File 1, array data for all genes changing significantly between the two populations; and Table 1, data for selected genes from all four populations], both of which expressed relatively high levels of CD9 and GCTM-2 antigen, was highly informative. This comparison showed that the P7 population expressed higher levels of a modest number of genes (271 in all at twofold or higher level) on the microarray, and that an even smaller number of genes were activated in P6 compared to P7 (47 at twofold higher levels). While the list of genes expressed more strongly in P7 contained many that have been associated with stem cell phenotype in previous studies, such as TDGF-1, DPPA4, ZFP42, DNMT3B, and TERF-1 [11] (see Additional File 1 and Table 1), many novel molecules potentially critical to the earliest stages of stem cell differentiation were identified through this comparison. A number of cell surface markers not previously associated with hESC were identified, including six transmembrane epithelial antigen of the prostate, supervillin, the chondroitin sulphate proteoglycans bamacan, versican, and opticin, SIDF, and Intm2a. In addition, a number of polypeptide regulatory factors and receptors are also identified, including neurotensin, adrenomedullin, and the endothelin receptor. It is also of interest that a number of genes involved in chromatin remodeling were expressed at highest levels in the P7 population and were downregulated as stem cell surface marker decreases.

**Table 1:** Microarray analysis of changes in expression level of selected genes across the four cell populations isolated by flow cytometry. Bold numerals indicate B-statistic greater than zero, italicized numerals indicate expression levels two-fold or higher in P7 versus other populations, and grey numerals indicate 0.5 fold or lower expression levels in P7 versus other populations.

**Gene Symbol**	**Accession number**	**Gene Name**	**fold change P7 V P4**	**B 7V4**	**fold change P7 V P5**	**B 7V5**	**fold change P7 V P6**	**B 7V6**
**ACVR2B**	NM_001106	activin A receptor, type IIB	0.89	-5.4	1.37	-2.7	*2.83*	**2.6**
**ADM**	NM_001124	adrenomedullin	*8.82*	**8.2**	*4.23*	**4.0**	*2.55*	**1.1**
**AFP**	NM_001134	alpha-fetoprotein	*1.61*	**1.8**	*2.06*	**1.7**	*1.88*	**0.4**
**BNC2**	AK001099	Homo sapiens cDNA FLJ10237 fis, clone HEMBB1000438.	*6.54*	**7.1**	*6.96*	**3.8**	*1.90*	**4.3**
**CALB1**	NM_004929	calbindin 1, 28 kDa	*9.32*	**3.3**	*6.36*	**5.5**	*2.48*	**4.6**
**CCNA2**	NM_001237	cyclin A2	*2.75*	**7.9**	*2.04*	**1.9**	1.46	**0.8**
**CCNE1**	M74093	cyclin E1	*1.98*	**7.6**	1.43	**0.8**	1.15	-1.7
**CD9**	AK025016	Homo sapiens cDNA: FLJ21363 fis, clone COL02986	*1.52*	**4.9**	*1.51*	0.0	1.35	-3.8
**CDH6**	NM_004932	cadherin 6, type 2, K-cadherin (fetal kidney)	0.36	**2.8**	0.43	**3.9**	0.39	**3.5**
**CEBPZ**	NM_005760	CCAAT-box-binding transcription factor	*6.15*	**0.7**	*2.68*	**0.6**	1.32	-5.6
**COL4A6**	U04845	collagen, type IV, alpha 6	0.36	**5.2**	0.44	**3.8**	0.47	**4.0**
**CRI1**	NM_014335	CREBBP/EP300 inhibitory protein 1	*2.35*	**2.5**	*2.39*	**2.3**	*2.83*	**4.4**
**CRYM**	NM_001888	crystallin, mu	*4.96*	**9.6**	*2.68*	**0.8**	1.37	-3.5
**CSPG2**	NM_004385	chondroitin sulfate proteoglycan 2 (versican)	*2.50*	**7.0**	*2.10*	-1.1	*2.85*	**2.1**
**CSPG6**	NM_005445	chondroitin sulfate proteoglycan 6 (bamacan)	*3.76*	**5.0**	*1.99*	**3.9**	*2.81*	**1.3**
**CTCF**	NM_006565	CCCTC-binding factor (zinc finger protein)	*1.61*	-3.2	*1.73*	**1.1**	1.15	-3.3
**DKFZP586A0522**	AF113007	DKFZP586A0522 protein	*5.35*	**5.5**	*4.76*	**2.9**	*3.84*	**4.6**
**DKK1**	NM_012242	dickkopf homolog 1 (Xenopus laevis)	0.33	-0.6	0.36	**5.0**	0.43	**3.6**
**DNMT3B**	NM_006892	DNA (cytosine-5-)-methyltransferase 3 beta	*3.14*	**4.7**	*2.68*	**0.9**	*1.97*	**1.1**
**DPPA4**	NM_018189	developmental pluripotency associated 4	*7.52*	**8.3**	*5.62*	**3.0**	*2.77*	**2.3**
**EDN1**	NM_001955	endothelin 1	0.45	**1.4**	0.72	**1.2**	0.97	-6.7
**EDNRB**	NM_000115	endothelin receptor type B	*3.05*	**6.9**	*2.27*	**0.3**	1.30	-1.1
**FBLN1**	NM_006485	fibulin 1	0.37	**5.0**	0.43	**2.9**	0.60	**1.1**
**FGFR1**	AK001052	Homo sapiens cDNA FLJ10190 fis, clone HEMBA1004753.	0.67	**1.2**	0.65	-1.5	0.59	**1.8**
**FGFR3**	NM_000142	fibroblast growth factor receptor 3 (achondroplasia, thanatophoric dwarfism)	0.39	**2.3**	0.37	**3.6**	0.42	**3.0**
**FLJ10036**	NM_017975	hypothetical protein FLJ10036 (zwilch)	*4.38*	**3.5**	*2.57*	**2.3**	*2.62*	**4.0**
**FLJ12787**	AK022849	hypothetical protein FLJ12787 (Src-associated protein SAW)	*3.16*	**6.3**	*2.04*	**1.9**	1.34	**1.6**
**FLT1**	NM_002019	fms-related tyrosine kinase 1 (vascular endothelial growth factor/vascular permeability factor receptor)	*4.23*	**2.0**	*3.10*	**3.4**	*1.67*	**3.5**
**FST**	NM_006350	follistatin	0.22	**4.9**	0.31	-0.5	0.42	**0.9**
**FZD7**	NM_003507	frizzled homolog 7 (Drosophila)	*3.76*	**2.0**	*1.71*	-2.1	1.23	-5.5
**GOLGB1**	NM_004487	golgi autoantigen, golgin subfamily b, macrogolgin (with transmembrane signal), 1	*2.10*	**6.2**	*1.77*	**1.6**	*2.83*	**2.6**
**GPC4**	NM_001448	glypican 4	*2.93*	**6.6**	*2.68*	**3.1**	*1.81*	**2.1**
**GREM1**	NM_013372	cysteine knot superfamily 1, BMP antagonist 1	0.57	-2.5	0.26	**6.0**	0.49	**1.8**
**HAT1**	NM_003642	histone acetyltransferase 1	*6.02*	**3.9**	*2.35*	**0.7**	1.17	-6.0
**HELLS**	AK021443	Homo sapiens cDNA FLJ11381 fis, clone HEMBA1000501.	*6.87*	**7.3**	*6.63*	**2.1**	*5.24*	**1.9**
**HESX1**	NM_003865	homeo box (expressed in ES cells) 1	*2.30*	**5.5**	1.25	-3.0	0.68	-0.8
**HSPCA**	D87666	heat shock 90 kDa protein 1, alpha	*4.14*	**0.7**	*2.71*	**0.6**	*2.62*	**5.4**
**HSPCB**	NM_007355	heat shock 90 kDa protein 1, beta	*3.71*	**1.6**	*2.04*	**3.2**	*3.63*	**2.3**
**KDR**	AF035121	kinase insert domain receptor (a type III receptor tyrosine kinase)	*2.53*	**4.8**	*1.94*	**0.1**	*1.94*	**1.9**
**LHX2**	NM_004789	LIM homeobox 2	0.04	**6.1**	0.17	**0.5**	0.48	**1.1**
**LIFR**	NM_002310	leukemia inhibitory factor receptor	*1.91*	**1.2**	*1.71*	**1.8**	*2.38*	**1.1**
**LOC57146**	NM_020422	promethin	*1.93*	**4.4**	*2.35*	**1.1**	*4.23*	**3.2**
**MSX1**	NM_002448	msh homeo box homolog 1 (Drosophila)	0.16	**6.5**	0.20	**1.3**	0.38	**0.9**
**NUP54**	NM_017426	nucleoporin 54 kDa	*4.17*	**3.5**	*1.96*	**1.3**	1.01	-7.3
**OPTC**	NM_014359	opticin	*4.79*	**3.2**	*4.11*	**0.4**	*2.62*	**0.6**
**PAX6**	NM_001604	paired box gene 6 (aniridia, keratitis)	0.04	**3.5**	0.13	**0.9**	0.29	**2.5**
**PB1**	NM_018165	polybromo 1	1.45	**1.8**	*1.54*	-0.4	*2.23*	**1.7**
**PODXL**	NM_005397	podocalyxin-like	*7.31*	**4.9**	*2.22*	**0.6**	1.38	-3.8
**SAS10**	NM_020368	disrupter of silencing 10	*3.20*	**6.4**	*3.58*	**2.6**	*3.20*	**3.4**
**SCAMP3**	NM_005698	secretory carrier membrane protein 3	0.48	**2.9**	0.51	**1.4**	0.57	**2.0**
**SDFR1**	NM_012428	stromal cell derived factor receptor 1	*3.66*	**6.1**	*2.50*	0.0	*1.51*	-0.8
**SFRP1**	NM_003012	secreted frizzled-related protein 1	*3.97*	**3.3**	*2.99*	**1.7**	*1.85*	-1.4
**SLIT1**	NM_003061	slit homolog 1 (Drosophila)	0.29	**2.6**	0.40	**1.5**	0.69	**0.8**
**SMARCA2**	NM_003070	SWI/SNF related, matrix associated, actin dependent regulator of chromatin, subfamily a, member 2	*4.72*	**6.8**	*3.58*	-0.3	*2.50*	**1.1**
**SMO**	NM_005631	smoothened homolog (Drosophila)	0.32	**5.4**	0.33	**2.5**	0.43	**2.4**
**SOX1**	NM_005986	SRY (sex determining region Y)-box 1	*2.69*	**0.9**	1.34	-2.7	*1.70*	**0.6**
**STEAP2**	AK026813	six transmembrane epithelial antigen of prostate 2	*4.72*	**9.4**	*2.73*	**1.2**	*2.85*	**3.5**
**T**	NM_003181	T, brachyury homolog (mouse)	1.04	-7.3	0.98	-7.3	1.01	-7.4
**TERF1**	NM_017489	telomeric repeat binding factor (NIMA-interacting) 1	*11.31*	**4.0**	*6.50*	**4.8**	*2.04*	**3.1**
**TGFBR3**	NM_003243	transforming growth factor, beta receptor III (betaglycan, 300 kDa)	0.37	**8.5**	0.39	**2.6**	0.52	**1.8**
**VEGFC**	NM_005429	vascular endothelial growth factor C	0.46	**0.7**	0.68	**1.5**	0.85	-3.7
**ZFP42**	NM_003422	zinc finger protein 42 (myeloid-specific retinoic acid-responsive)	0.37	**5.1**	0.56	**2.3**	0.80	-2.4

Analysis of genes upregulated as stem cell surface marker expression decreases pointed to neural differentiation as a key pathway undergoing activation. Amongst the most strongly induced genes in the early stages of differentiation are transcription factors characteristic of early neural lineages such as Pax-6. The early activation of these neuralising factors may account for the tendency of hESC cells to undergo "spontaneous" neural differentiation under these conditions of culture [34]. At the same time, the expression of bone morphogenetic proteins and Wnt antagonists such as noggin, chordin and dickopf, is activated. These molecules are known as neuralising factors from animal embryology [35] and they and other related molecules are involved in induction of the formation of the nervous system. Moreover, several surface markers of these early differentiating populations were identified, including betaglycan, smoothened, cadherin 6 type 2 and FGFR3. These cell surface molecules, expressed only at low levels in P7, may serve to mark the early differentiating cells from the most primitive stem cell population.

We directly compared our results with the only other study in the literature to examine gene expression in hESC sorted by flow cytometry. Enver et al. [36] compared gene expression in hESC positive for the cell surface glycolipid antigen SSEA-3 (SSEA-3^+^) to SSEA-3 negative (SSEA-3^-^) cells. Of the 468 genes differentially expressed by their Affymetrix analysis, 397 could be identified on our Compugen array. 82 genes that were significantly elevated (B score>0 and >2 fold up regulated) in our P7 population versus P6, P5, or P4, were also elevated in SSEA-3 positive cells compared to SSEA-3 negative cells, including TDGF1, DNMT3B, FLJ12505, GPC4, BMPR1A, ADM, CALB1, KIT, and TERF1, all identified in previous array studies of hESC. Further analysis showed that there was greater concordance between the set of genes differentially expressed in P7 versus P4 and SSEA-3^+ ^cells versus SSEA-3^- ^cells than between the other sets of comparisons that we carried out. Thus 75 genes are more highly expressed in both P7 versus P4 as well as SSEA-3+ versus SSEA-3^- ^cells (~19% of P7 versus P4 gene list), while 36 genes are more highly expressed in both P7 versus P5 and SSEA-3+ versus SSEA-3^- ^cells (~7% of P7 V P5 genelist), and only 28 genes are more highly expressed in both P7 versus P6 and SSEA-3+ versus SSEA-3^- ^cells (approx 9% of P7 V P6 gene list). In summary, our study, which compared hESC populations expressing different levels of stem cell antigens, identified a different set of regulated genes to this previous study, which compared cells expressing stem cell antigens to cells that did not. As expected, the greatest overlap between the two studies was seen in our comparison of cells expressing the highest level of stem cell antigens to the negative cells.

### QRT-PCR confirms coordinated downregulation of pluripotency genes and concomitant activation of transcription factors associated with induction of extraembryonic endoderm and neurectoderm

**Figure 5:**
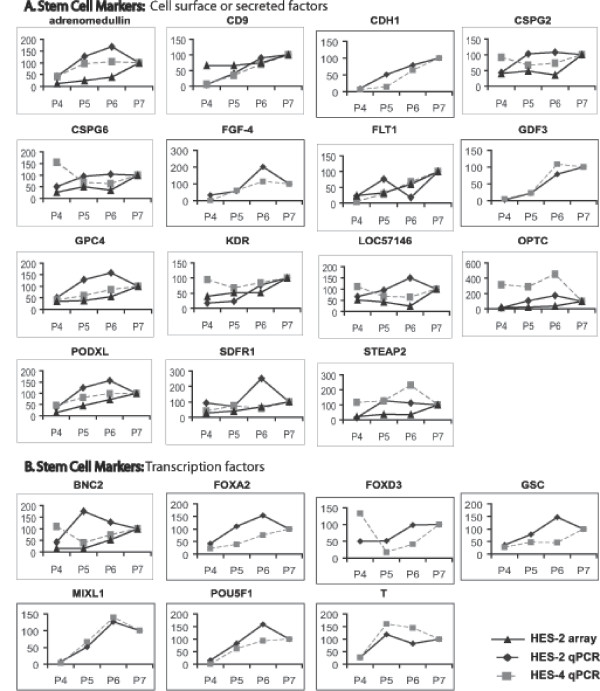
Relative gene expression levels of combined array and QRT-PCR analyses of P4, P5, P6 and P7. Stem cell markers are presented relative to P7 (set at 100). A, stem cell markers: cell surface or secreted factors. B, stem cell markers: transcription factors.

**Figure 6:**
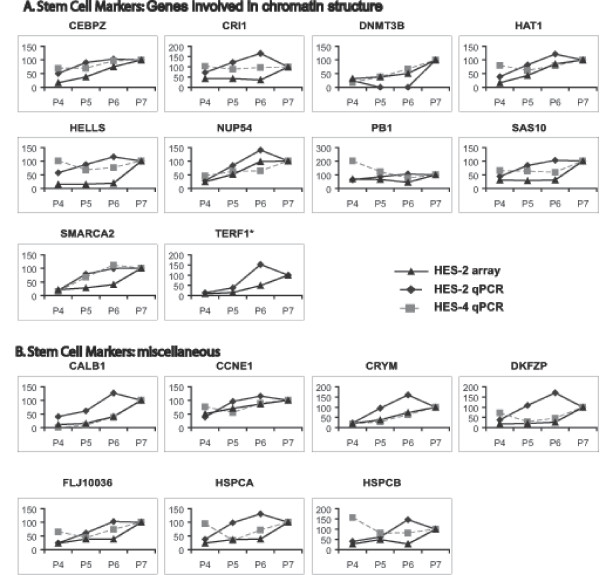
Relative gene expression levels of combined array and qPCR analyses of P4, P5, P6 and P7. Stem cell markers are presented relative to P7 (set at 100). A, stem cell markers: genes involved in chromatin structure. B, stem cell markers: miscellaneous genes.

**Figure 7:**
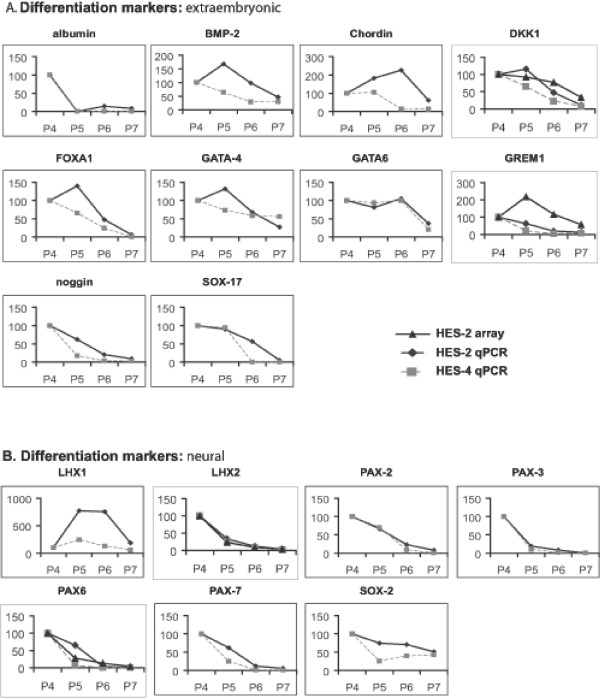
Relative gene expression levels of combined array and QRT-PCR analyses of P4, P5, P6 and P7. Differentiation markers are presented relative to P4 (set at 100). A, differentiation markers: extraembryonic. B, differentiation markers: neural.

**Figure 8:**
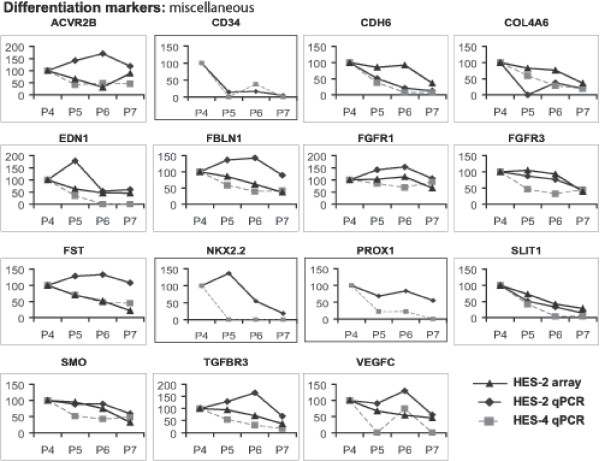
Relative gene expression levels of combined array and QRT-PCR analyses of P4, P5, P6 and P7. Differentiation markers are presented relative to P4 (set at 100). A, differentiation markers: miscellaneous cell surface or secreted factors. B, differentiation markers: miscellaneous transcription factors.

The results described above were confirmed and extended by QRT-PCR using ABI Microfluidic card analysis of 96 genes selected in part from those identified in the array analysis, and some key stem cell maintenance factors and differentiation markers that were not represented on the array or failed to read out. Two separate hESC lines were immunologically fractionated as described above, RNA was prepared, cDNA was synthesised, and analysed for expression of the genes shown. Downregulation of transcript levels of a number of known and novel markers of the pluripotent state was confirmed (Figures 5 &6). Transcripts for CD9, which was used for flow cytometry sorting of the population, showed the expected reduction, as did many other markers of the pluripotent state. The QRT-PCR analysis highlighted that genes that are activated early on after loss of stem cell markers include genes expressed in extraembryonic endoderm and neural genes (Figures 7 &8). Thus, the transcription factors GATA-4, GATA-6, and FOXA1 are all activated, as is the Wnt antagonist dickopf; these transcription factors are critical to the differentiation of primitive endoderm in the mammalian embryo, and dickopf is secreted by this tissue. Note that in Figures 5, 6, 7, 8, the use of a value of 100 on the y-axes to indicate expression levels relative to the P7 or P4 population (depending on which was higher), and based on either array or QRT-PCR, enables comparison of the data but also compresses its display, and can thus obscure significant differences where the overall change in expression is large. For example, the relative fold change in Pax-6 expression for HES-2 in the array data was 3.5 (P6), 7.4 (P5) and 27 (P4) relative to P7, and the corresponding data for QRT-PCR 5.0, 88, and 135 fold. Thus there is clearly activation of Pax-6 expression in P6 versus P7 by either technique, but the graphic display of this in Figure 7 is influenced by the massive overall change between P7 and P4.

The analysis also confirmed the induction of BMP and TGF-beta antagonists including chordin, noggin, gremlin, and follistatin. These genes are transcribed in tissues involved in patterning in the mouse postimplantation embryo, the node and the anterior mesendoderm, and they function to drive commitment of pluripotent cell populations to neural fates. Possibly as a consequence of the production of these anteriorising factors, transcription factors characteristic of neurectoderm are activated during differentiation prior to overt loss of stem cell marker expression (Figure 7B), and the transcription factors characteristic of mesendodermal lineages (T, MixL-1 and goosecoid) are switched off. The picture is consistent with the early commitment of ES cells to the extraembyonic lineage and the subsequent elaboration of factors from these cells and other cell types that drive neural commitment. It is important to note that the expression of most of these lineage specific transcription factors begins to rise well before extinction of expression of markers of pluripotency.

Expression of certain surface markers for ES and differentiated cells was also confirmed (Figures 5A &8A), as was the downregulation of a number of genes involved in chromatin structure (Figure 6A).

## Discussion

The hESC phenotype has been defined at the immunological, transcriptional, and biological levels. This study has shown that flow cytometric sorting based on quantitative levels of expression of two surface markers, the GCTM-2 antigen and CD9, allows fractionation of the hESC population into subsets expressing varying levels of pluripotency genes. The GCTM-2 antigen is not exclusive to primate pluripotent stem cells, but its expression is informative of stem cell status in a restricted context. The function of this proteoglycan is unknown. It is of interest that our array studies found that several known chondroitin sulphate proteoglycan core proteins were highly expressed in stem cells and rapidly downregulated during the early stages of differentiation. It is now appreciated that, like the better-studied heparan sulphate proteoglycans, chondroitin sulphate proteoglcyans can function to present growth factors to cells [37]. Thus these chondroitin sulphate proteoglycans may represent an important component of the hESC microenvironment. CD9 is a tetraspannin protein thought to function in organizing integrins and other receptors at the cell surface. There is some evidence to implicate the molecule in ES cell maintenance in the mouse [38]. CD9 transcript levels, measured by microarray or Q-RTPCR, correlated well with levels of the protein as determined by flow cytometry.

For this study, we cultured hESC in the presence of a fibroblast feeder cell layer and fetal calf serum, using mechanical dissociation to passage the cells. This method provides for long term support of diploid populations of hESC. Other culture methodologies may give rise to the appearance of chromosome abnormalites in hESC cultures [39-41]. Since these abnormal cells often have altered growth and differentiation properties [36,41], which reflect changes at the level of gene transcription, and since it is not clear at what stage during their emergence the phenotypic changes occur, we conducted our study using a culture method that reliably maintains the diploid state. It will be of interest to carry out this analysis on cells grown under different conditions. Preliminary data indicate that there is a similar continuum of marker expression in cultures grown in FGF-2 and proprietary serum replacement.

The use of several surface markers enabled separation of the hESC population into subsets of cells at various stages of differentiation, as indicated by their expression of stem cell markers and lineage specific transcription factors. Perhaps because the cell populations we examined are so closely related, relatively few genes showed significant changes in transcript levels across the cell populations studied, and some novel candidate surface markers and stem cell regulators were identified. Novel surface markers will facilitate identification of stem cell subpopulations and early differentiated cells. Ligands such as endothelin and their cognate receptors may have roles in stem cell regulation.

In addition to these surface molecules and receptors and ligands, it is notable that a number of genes with known or suspected function in chromatin remodeling were downregulated during the early phases of stem cell differentiation. Although chromatin remodeling is clearly important during differentiation, and chromatin remodeling proteins are thought to be critical components of oocyte cytoplasm for the reprogramming process that occurs in the donor nucleus during cloning by somatic cell nuclear transfer, previous studies have not reported an overall downregulation of these molecules during stem cell differentiation. The study of Boyer et al. [27] identified chromatin remodeling complexes as targets of Oct-4. It is possible that chromatin plasticity is an essential feature of the pluripotent state and that expression of remodeling factors is therefore important to stem cell maintenance. In Arabadopsis, maintenance of the pluripotent stem cell population of the apical meristem depends on expression of both specific transcription factors and a set of chromatin remodeling factors [42]. Deficiencies in chromatin remodeling factors (ISWI) lead to loss of germline stem cells in Drosophila, due to defects in the response of the cells to signals from the niche and a loss of suppression of differentiation [43]. Recently results based on quantitative in vivo imaging and biochemical examination of chromatin proteins in mouse ES cells strongly argues that a highly dynamic state of architectural chromatin proteins is associated with pluripotency [44].

It is clear from these results that stem cell maintenance factors are co-expressed at early stages of the differentiation process along with a number of transcription factors characteristic of commitment to extraembryonic endoderm, mesendoderm, and neural lineages. As expression of hESC markers decline, markers of neural and extraembryonic lineages remain on or increase, whereas mesoderm marker expression decline. The coexpression of stem cell and lineage specific transcription factors was first described in stem cells of the hematopoietic lineage [24,25], where the phenomenon has come to be known as lineage priming. In essence the concept states that stem cells, rather than completely repressing lineage specific commitment genes, express these genes at low levels pending internal or external signals that activate specific differentiation programs fully. In our case, neuralising signals, in part from extraembryonic endoderm, may function to drive this outcome. Although the lineage priming model has been questioned, on the grounds that the cells expressing lineage specific markers might represent cells already committed to differentiation, marking of hematopoietic stem cells using cre recombinase driven by the myeloid lineage specific promoter for lysozyme, showed that marked cells which activated the gene were capable of long term renewal and multipotent differentiation [45].

Ultimately, it will be critical to define the developmental potential of these various subpopulations of hESC. Currently, technical limitations restrict our ability to examine this question in detail. Isolation and clonal analysis of single hESC following flow cytometry sorting is problematic at present, due to low cell survival, but our preliminary results indicate that the cells with reduced surface marker expression, shown here to display activation of specific lineage commitment programs at the transcriptional level, are clearly capable of maintaining a stem cell phenotype. It should be noted that although we used two independent markers to fractionate the cell population, the fractions we obtained are still likely to be heterogeneous. Andrews and colleagues [36] analysed hESC cultures and separated them into populations which were positive or negative for the surface marker SSEA-3; they concluded that SSEA-3 negative cells were an intermediate population between the pluripotent state and fully committed cells. Whether the cells with the highest levels of surface marker and pluripotency gene expression are already primed at the transcriptional level will require more refined analysis of the population, but some lineage specific factors are expressed at appreciable levels even in this population.

These results have implications for interpretation of biochemical and molecular studies on hESC, and for practical approaches to their propagation and manipulation. Clearly interpretation of molecular studies of hESC should take the heterogeneity of the cultured cell population into account. The presence of hESC subpopulations that are apparently primed for commitment to different cell fates, at least at the transcriptional level, suggests that the large scale production of pure populations of pluripotent cells or committed progenitors will require a much better understanding of the dynamics of these early stages of commitment and the interactions of the various cellular subpopulations that are present in the culture. Moreover, these results point to a special role for ES cells in fundamental research aimed at understanding the cellular and molecular basis of fate determination. Because they may be grown in large numbers and readily fractionated by immunological means into populations at very early and defined stages of determination, hESC may represent an excellent model system for studying how lineage commitment occurs in a stem cell population and for defining the molecular events that accompany loss of pluripotency.

## Conclusion

Human embryonic stem cell cultures contain a heterogeneous mixture of cells representing a continuum along a differentiation hierarchy. Cells in this hierarchy co-express stem cell markers along with transcription factors characteristic of specific differentiation lineages.

## Methods

### Cell Culture

hESC lines HES-2, -3, and -4 were grown as previously described [5], using serum containing medium, mouse embryonic fibroblast feeder cell support, and mechanical dissection of colonies for subculture. This methodology was used for hESC culture because in our hands this approach provides for long-term maintenance of pluripotent stem cells with a normal diploid karyotype.

### Indirect immunofluorescence

Triple indirect immunofluorescent staining for GCTM-2, Oct-4 and TG30 was carried out using isotype specific secondary antibodies. Oct-4 was detected with anti-mouse IgG2bAF568 (1:1000), TG30 with anti-mouse IgG2aAF488 (1:1000) and GCTM-2 with biotinylated anti-mouse IgM (1:125, Dako) followed by streptavidin AF350 (1:1000, all from Molecular Probes). Double or triple stained slides were counterstained with DAPI and mounted in ProLong Antifade (Molecular Probes, OR, USA).

### Immunohistochemistry

Normal human kidney tissue was obtained from the uninvolved pole of renal carcinoma nephrectomy specimens (approved by the Monash Medical Centre Human Research Ethics Committee). Sections (4 μm) of formalin-fixed, paraffin embedded tissue were dewaxed, hydrated in PBS and blocked in 10% normal sheep serum and 10% foetal calf serum in PBS for 30 min. Sections were incubated overnight with GCTM-2 or PHM-5 [46] antibody at 4°C, washed (x3) in PBS; endogenous peroxidase was inactivated in 0.6% H_2_O_2 _in methanol for 20 min, and the sections washed in PBS. Sections were then incubated with either horseradish peroxidase (HRP)-conjugated goat anti-mouse IgG (Dako, Glostrup, Denmark) (for detection of PHM-5) or goat anti-mouse IgM (Serotec, Oxford, UK) (for detection of GCTM-2) in 5% normal sheep serum and 3% bovine serum albumin in PBS for 40 min, washed (x3) in PBS, incubated with complexes of HRP-conjugated mouse anti-HRP IgG complexes (Dako), washed (x3) in PBS and developed with the diaminobenzidine substrate (Sigma-Aldrich, Castle Hill, NSW, Australia) to produce a brown colour.

### Mammalian expression of recombinant podocalyxin and immunoblot analysis

Preliminary attempts to express recombinant human podocalyxin in mouse STO cells resulted in the production of a protein immunoreactive with monoclonal antibodies specific for human podocalyxin, but this protein was much smaller than the canonical form, suggesting either premature termination of transcription or translation, partial degradation, or incomplete glycosylation. We reasoned that a mouse cell line that normally expresses the protein might produce mature full-length human podocalyxin when transfected with recombinant cDNA. Mouse M15 cells, derived from embryonic mesonephros [47], were grown in Dulbecco's Modified Eagle's Medium (high glucose formulation) supplemented with 20% foetal calf serum, penicillin and streptomycin and L-glutamine. For transfection, M15 cells were plated on T-75 flasks the day before to allow 50–80% confluency at the time of transfection. An hour before transfection, media was replaced with serum-free, supplemented media. For each transfection, 30 μL Fugene 6 was diluted in 470 μL of serum-free media. DNA at a ratio of (v/w) 3:1 (Fugene 6: DNA) was then added and incubated for 15 mins at room temperature. The mixture was added dropwise to the cells which were incubated at 37°C with 5% CO_2_. 5 hours after transfection, the media was replaced with serum-containing, supplemented media. Cells were lysed 48 hours post-transfection using lysis buffer containing 10 mM Tris-HCl buffer (pH 7.0), 1% Triton X-100 detergent and 1 mM EDTA in water. 1 mM phenylmethanesulfonyl flouride diluted in isopropanol was added to the lysis buffer prior to use. Protein lysate samples were separated in a 6% reducing SDS-PAGE gel and transferred for 1 hour at 75 mAmps onto a PVDF membrane. Blots were blocked in 5% skim milk powder in PBS-Tween overnight and incubated with the monoclonal antibodies GCTM2, podocalyxin (PHM-5) and TG343 for 1 hour. Washed blots were then incubated with the secondary antibody anti-mouse Ig-HRP for an hour and the blots were developed with ECL.

### FACS Analysis and Cell Sorting

For analysis of co-expression of Oct-4 and surface markers, harvested hESC were dissociated into single cell suspension by trituration and fixed with 100% methanol. Cells were then stained with a mixture of mouse IgM GCTM-2, mouse TG30 (anti-CD9) IgG2a and mouse Oct-4 IgG2b or a mixture of class matched negative control antibodies. Binding of primary antibodies was detected by incubation with biotinylated rabbit anti-mouse IgM, followed by a mixture of goat anti-mouse IgG2a-AF488, goat anti-mouse IgG2b AF647 and streptavidin-PE. Samples were assayed on a flow cytometer (FACS Vantage-SE Diva, Becton Dickinson). Cells were gated initially using forward and right angle light scatter and AF488, AF647 and PE fluorescence signals were collected. hESC cells analysed via the above method were compared to single color controls for TG30, GCTM-2 and Oct-4 and parallel analyses examined live, non-fixed, human ES cells for the presence of the cell surface markers GCTM-2 and TG30. Co-incubation of the primary antibodies with each other and with fixed or non-fixed human ES cells did not affect the percentages of cells displaying immunofluorescence for each antibody.

For preparative isolation of discrete cell populations for RNA analysis, unfixed hESC were harvested and stained in solution for GCTM-2, TG30 (CD9) and Thy1.2-PE (to gate out any remaining mouse embryo fibroblasts) as above, except that the secondary antibodies used were goat anti-mouse IgG2a-AF488 (Molecular Probes, Oregon, USA) and goat anti-mouse IgM AF647 (Molecular probes, Oregon, USA). Cells were sorted four ways into microfuge tubes (P4, P5, P6 and P7, see Figure 4a above) using the FACSVantage-DIVA (BDBiosciences). Sorted cells were initially gated using forward and side scatter, followed by the removal of clumps and doublets by gating on single cells (FSC-A vs. FSC-H), and the removal of MEF feeder cells using negative selection for Thy1.2-PE.

### Microarray analysis

#### RNA amplification and target labelling

Total RNA from the sorted fractions described above was isolated using Trizol and linearly amplified using the messageAMP aRNA kit (Ambion) yielding a minimum of 10 micrograms of amino-allyl labeled anti-sense aRNA. The quantity and integrity of these aRNAs was compared via running each sample on a bio-analyser RNA micro-fluidic chip (Agilent) prior to labelling. 5 micrograms of each aRNA sample was then labelled by covalent linking Cy5- or Cy3-labelled UTP (Amersham). Finally the labelled material was hydrolysed and used for hybridisation.

#### Array fabrication and generation

The arrays used were obtained from the SRC Microarray Facility, University of Queensland (ARC Centre for Functional and Applied Genomics) and comprised 17260 human gene-specific oligonucleotides (Compugen) spotted onto epoxy-silane coated slides (Fullmoon). Arrays were hybridised for a minimum of 16 hours at 45°C using previously described conditions [48].

#### Image Analysis, normalisation and analysis

Hybridised slides were washed, dried and scanned in a 600B array scanner (Agilent). The images were analysed with Imagene 5.5 (BioDiscovery Inc) to determine mean foreground and background for both channels. All primary data, including images was then imported into an in-house installation of the comprehensive microarray relational database, BASE [49].

The raw data from each hybridisation was compiled into an experiment and subjected to print tip intensity independent Lowess normalisation using the R statistical software from the LIMMA package [50]. This normalisation is implemented within BASE using scripts developed by Ola Spjuth of the Linnaeus Centre for Bioinformatics [51]. Gene lists, along with MA-plots and box plots showing the normality of the data are available via the BASE database cited above (login Laslett 2006, Password: HES).

#### Experimental design

The four FACS sorted populations were compared to one another in a boxed experimental design where each sample is compared to the other in at least triplicate; a dyeswap was included to account for dye bias. Differential expression was defined using a robust statistical method rather than simple fold change. All genes were ranked using the B statistic method where both fold change and variance of signals in replicates is used to determine the likelihood that genes are truly differentially expressed. A threshold in the B statistic of 0.0 was adopted as genes with a B score>0 have a >50% probability of being truly expressed [52]. This analysis was executed using the Bio-conductor package that has been implemented as a plug in tool in BASE, where the actual B values may be found.

Data deposition note: The full array dataset is available from Gene Expression Omnibus (GEO, Accession Number GSE4020) [53].

#### Q-RT-PCR confirmation of gene expression patterns

We used the ABI Microfluidic Card system for quantitative RT-PCR validation of patterns of gene expression. We designed a 384 well format card enabling assay of 96 target genes on four samples. The card incorporates proprietary validated RT-PCR primers for key genes identified in our original studies as strongly up- or down-regulated during early phases of hESC differentiation, plus a number of classical hESC genes and a number of genes characteristic of early differentiation pathways. To carry out analyses, total RNA (1 μg) was isolated, reverse transcribed, and 100 ng introduced into the gene card in PCR mastermix. Amplification was carried out in the ABI Prism 7900 HT system at the Australian Genome Research Facility Melbourne Node, and analysed using the comparative C_T _method using the proprietary sequence detection software.

The comparison of expression levels was carried out with reference calibration to the population of cells with the highest level of stem cell marker expression.

## Abbreviations

ES – embryonic stem

hESC – human embryonic stem cells

QRT-PCR – quantitative reverse transcriptase polymerase chain reaction

## Authors' contributions

ALL carried out many of the experiments and helped design the study, SG and BG carried out microarray analyses, LS and SH carried out experiments to show that antibody TG30 recognizes CD9; AL and DN-P carried out the experiments on the relationship of podocalyxin and the GCTM-2 antigen; SW helped carry out and analyse QRT-PCR, DH developed the flow cytometry separation methodology, and MFP conceived the study and wrote the manuscript. All authors read and approved the final manuscript.

## Supplementary Material

Additional File 1 Annotated array data comparing gene expression between the P6 and P7 populations. The data provided in this table show in detail all genes that change significantly between the two populations P6 and P7 Click here for file
